# Segmentation of Heavily Clustered Nuclei from Histopathological Images

**DOI:** 10.1038/s41598-019-38813-2

**Published:** 2019-03-14

**Authors:** Mahmoud Abdolhoseini, Murielle G. Kluge, Frederick R. Walker, Sarah J. Johnson

**Affiliations:** 1 The University of Newcastle, School of Electrical Engineering and Computing, Callaghan, NSW 2308 Australia; 2 The University of Newcastle, School of Biomedical Sciences and Pharmacy, Callaghan, NSW 2308 Australia; 3 The Hunter Medical Research Institute, New Lambton, NSW 2305 Australia

## Abstract

Automated cell nucleus segmentation is the key to gain further insight into cell features and functionality which support computer-aided pathology in early diagnosis of diseases such as breast cancer and brain tumour. Despite considerable advances in automated segmentation, it still remains a challenging task to split heavily clustered nuclei due to intensity variations caused by noise and uneven absorption of stains. To address this problem, we propose a novel method applicable to variety of histopathological images stained for different proteins, with high speed, accuracy and level of automation. Our algorithm is initiated by applying a new locally adaptive thresholding method on watershed regions. Followed by a new splitting technique based on multilevel thresholding and the watershed algorithm to separate clustered nuclei. Finalized by a model-based merging step to eliminate oversegmentation and a model-based correction step to improve segmentation results and eliminate small objects. We have applied our method to three image datasets: breast cancer stained for hematoxylin and eosin (H&E), Drosophila Kc167 cells stained for DNA to label nuclei, and mature neurons stained for NeuN. Evaluated results show our method outperforms the state-of-the-art methods in terms of accuracy, precision, F1-measure, and computational time.

## Introduction

Segmentation of cell nucleus from histopathological image, has been a focus of clinical practice and scientific research for more than half a century^1^. Automated nucleus segmentation is fundamental to cell counting, movement tracking, and morphological study, such as feature extraction and classification. This leads to a valuable insight into the cell features and functionality which result in early diagnosis of diseases such as breast cancer and brain tumour.

Despite considerable progress in automated segmentation, it remains a challenging task to separate a large clump of nuclei and delineate their boundaries with a high accuracy and speed. Under/over-segmentations happen in the presence of heavily clustered nuclei, due to the variability and complexity of data caused by noise, uneven absorption of stains, different cell types, etc.

The main role of every segmentation method is to separate an image foreground from background. These methods can be categorized into three general groups: 1- *optimization-based* in which an energy/cost function is maximized/minimized, e.g. active contours^2,3^, level set^4,5^, global minimizers^6^, graph-based^7^; 2- *machine-learning-based* in which a machine/network is trained to recognize features, in particular deep convolutional neural networks have drawn many attention recently^8–12^; 3- *threshold-based* in which a set of thresholds are found, e.g. iterative-based^13^, information-theoretic-based^14–16^, histogram-based^17–19^.

Recently, optimization methods have been employed to introduce new segmentation techniques. A level set method guided by seed points has been proposed by Husham *et al*.^4^. The seeds were extracted from the centroids of the objects via Otsu’s thresholding method and some basic morphological operators. The objective function of Otsu’s multilevel thresholding has been maximized based on a particle swarm optimization to achieve the optimum thresholds^18^. Then, breast cancer images were quantized through these thresholds to segment the nuclei. A semi-automatic optimization model has been proposed by Law *et al*.^20^ to segment multiple images. A user marks some sample pixels of different classes or objects existing inside one or more images to make labelled images. Then a classifier is trained based on the labelled images to classify other pixels inside the images or any other set of images which share the same features. Overall, the optimization-based approaches are problem dependent, computationally expensive, and must be initialized carefully through predefined parameters^1,12,21^.

Machine learning approaches have recently become popular in this area. A deep convolutional neural network (CNN) was learned by Xing *et al*.^12^ using three annotated datasets (brain tumour, pancreatic neuroendocrine tumour, and breast cancer), and a region growing approach was employed to generate binary maps of images. Then, a deformable model was implemented via learned shape dictionaries to segment the nuclei and delineate the boundaries. A very similar CNN was developed by Kumar *et al*.^11^ to generate ternary maps (three class of background, foreground, and boundary) of the image datasets stained for hematoxylin and eosin (H&E). A colour normalization pre-processing and an anisotropic region growing post-processing were also employed to delineate nucleus boundaries and refine their results. Although some manually annotated datasets (including ground truth data) are publicly available^11,22^, each dataset is stained with a specific dye to target a specific protein within the cells. Therefore, learning a machine/network to segment a desired object requires preparing a sufficiently large manually annotated dataset of the object. This tedious and painstaking task impedes the development of machine learning approaches applicable to images of various cell types which are stained for diverse proteins and produced by different labs.

Thresholding methods have the advantage of requiring no training, however they must be adaptive to become sufficiently discriminant for the heterogeneous intensity of microscopy images. Locally adaptive thresholding is a technique to tackle the heterogeneity of intensity by defining a specific threshold for each local region. One way is to define the regions using a moving window. A fixed size window moves across the image and divides it into small regions. Both window size and moving distances must be carefully assigned to avoid missing objects or adding noise via this approach^23^.

Microscopy images often include some dense area covered with clumps of nuclei that appear in the extracted foreground. Delineating the boundary of each individual nucleus inside the clustered object is the next task. A splitting technique based on size-constrained clustering has been introduced by Al-Kofahi *et al*.^24^. This method is computationally expensive and not feasible with large data, therefore a local-maximum clustering has been employed to reduce its complexity^25^. This requires a resolution parameter which is sensitive and fails in the presence of heavily clustered objects (see the Results section for details). Breast cancer tumours have been identified by Fatichah *et al*.^26^ via amplification of desired colour vectors of foreground/objects, and weakening of undesired vectors of background using the Gram-Schmidt method. Then, a cluster validation algorithm was proposed to separate the touching nuclei based on the Bayesian method, however many cells could not be separated^26^.

The watershed algorithm is frequently exploited in literature to split clustered objects^27–29^. In the classical approach, watershed is applied to the negative distance map of a binary array. This algorithm breaks the map into subregions by ‘flooding’ from regional minima until reaching ‘dams’ (separating lines). There are more regional minima than the actual number of the nuclei in a clump, which cause oversegmentation. The regional minima have been replaced by some detected shape markers/seeds to overcome this problem in recent approaches^23,30–32^. The shape markers have been employed to compute a marking function which is an ‘outer distance map’ concentrated around the markers^31,32^. There would be a nucleus for each marker, therefore a desired nucleus detection/segmentation is achievable through a correct set of markers. However, this can only be accomplished if several predefined parameters are carefully adjusted in these methods. Post-processing based on the nucleus model (shape and size) is another promising technique to deal with the oversegmentation problem^33–36^. Each type of cell has a particular shape (mainly oval) and its size lies within a particular range, therefore a proper model can be defined based on these features. Achieving the best possible model by rejoining the oversegmented objects is carried out in this post-processing.

**Figure 1:**
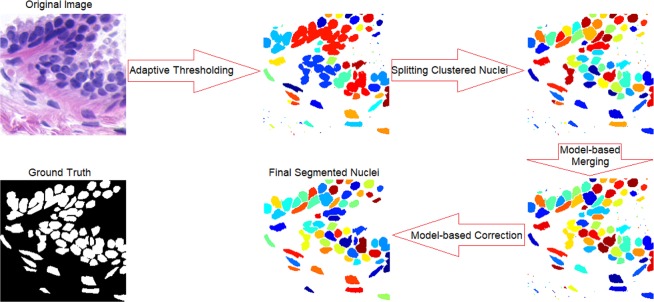
A flowchart of four main stages of our method. The original image and its ground truth are provided in the UCSB dataset of breast cancer^38^.

In this paper, we propose an automated algorithm to segment the cell nuclei from histopathological images. We first introduce a novel algorithm of locally adaptive thresholding on watershed regions. Then, we split clustered nuclei via a novel technique based on watershed and multilevel thresholding. We finalize our algorithm with two post-processing steps of model-based merging and correction (Fig. 1). A benefit of our algorithm is its simplicity of application with no training required and only a single parameter, minimum object size, required to be set by the user. Experimental results show our algorithm is very successful even on images with heavily clustered area/volume, and superior to the state-of-the-art methods. The rest of this paper is organized in three sections: Method in which our approach is described in detail, Results in which the experimental results are presented and compared with the state-of-the-art methods, and Discussion which concludes this paper.

## Method

### Local thresholding on watershed regions

Image pixels/voxels can be classified into two classes of foreground and background via many reported methods in literature^14^. However, it is rarely sufficient to recognize the foreground throughout a heterogeneous intensity image using a single threshold. In other words, a single threshold might be suitable to recognize the foreground in some part of an image, but unsuitable for other parts of the image. To overcome this problem, local thresholding is required to treat different parts of an image independently. In the following, we propose a novel local thresholding using watershed regions.

At the beginning of our segmentation process, 2D/3D microscopy images, fluorescent or brightfield, are converted to 2D/3D grey scale images in which the foreground is darker (has lower intensity) than background. Next, a global thresholding is applied to locate general objects throughout the image. We have used Otsu’s method^19^ to define both global and local thresholds, since it is one of the fastest and simplest methods available.

**Figure 2:**
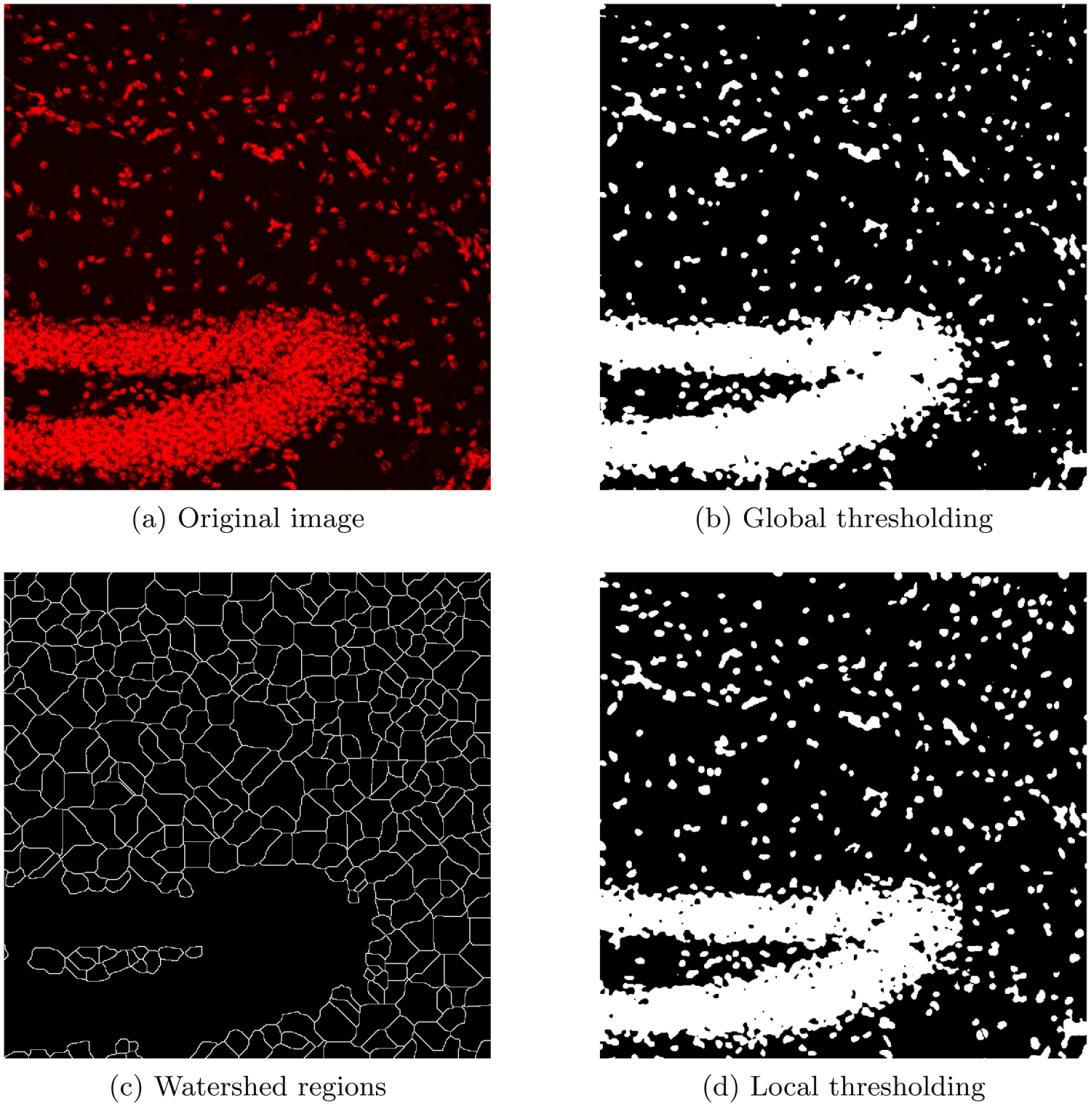
Local thresholding on watershed regions.

Let the binary array after global thresholding be denoted by *G*. We directly apply the watershed algorithm to a complement of *G* (with ‘0’s as objects and ‘1’s as the background) to create watershed lines/surfaces which divide the image into subregions each of which are formed around an object (Fig. 2c). These watershed lines/surfaces go through empty spaces between the objects while keeping the equal distances from their boundaries. Local regions created in this way cover all the image area/volume and are ready to be thresholded individually. Note that in this step we are not applying the watershed algorithm to split/segment the objects but rather to define regions for local thresholding.

A minimum object parameter, *ω*_min_, must be set before running our algorithm. *ω*_min_ should be set around the minimum nucleus size which is desired to be segmented. We threshold each local region that includes an object with a size greater than 0.1 × *ω*_min_. This reduces complexity by avoiding the local thresholding in empty regions, however this could be skipped if there was significant intensity differences and instead threshold all the regions. The output of local thresholding is denoted by *B*. Fig. 2 illustrates the globally thresholded array, the watershed regions derived from it, and the output of the local thresholding on these regions. It can be seen in Fig. 2, many missing nuclei after the global thresholding, are fully segmented via the local thresholding. We note that cell clusters can be seen in the output (Fig. 2d). For the next step, we propose an approach to split these clustered nuclei and outline their boundaries.

### Splitting clustered nuclei

Very dense areas covered with heavily clustered nuclei are sometimes included in histopathological images. They look like a very big object made of many attached nuclei. We aim to split these nuclei and outline the boundary of each individual nucleus. Watershed is a very powerful tool to accomplish this goal. As mentioned, classical watershed is applied to the negative distance map to break it into subregions. The distance map is an array in which every ‘0’ pixel of a binary array is reassigned to a value via a distance transform. For each ‘0’ pixel, this value equals its minimum distance to the closest ‘1’. Distances can be defined in several ways such as: Euclidean (the most common), quasi-Euclidean, cityblock, and chessboard, in the distance transform^37^. The distance transform is applied to the complement of the binary array (in which ‘0’s form the desired objects) and produces the distance map. Each subregion includes one regional minimum, separates it from others, and outlines an independent object. In other words, after applying watershed to the negative distance map, each regional minimum of the map produces one separate object split from the large clustered object. Since there are always more regional minima than the actual number of the touching nuclei, oversegmentation will occur. Recently proposed techniques addressing this problem are mentioned in the introduction, and their pros and cons are discussed. Unlike the aforementioned techniques, we propose a method that leverages the image intensity and incorporates it into the distance map. This significantly improves the recognition of the nucleus outlines.

**Figure 3:**
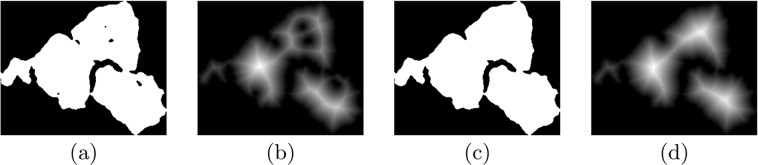
Positive effect of filling small holes. (**a**) Binary array including small holes. (**b**) Distance Map of (**a**), intensity is dispersed due to the small holes. (**c**) Binary array without small holes. (**d**) Distance Map of (**c**), shows the desired map with high intensity at the centres of the nuclei and low at the boundaries.

Before explaining our splitting method, note that every hole (connected ‘0’ pixels), no matter how small it is (even one pixel), inside the binary array can significantly change the distance map. Since they become short cuts of ‘1’s in the complement binary array and shorten distances which are supposed to be calculated from edges. Therefore filling small holes is necessary before the splitting process. However, we are interested in keeping the big holes, since they most likely locate the places where splitting must occur. Therefore holes less than the average size of all the holes in *B* are considered small, and filled. Fig. 3 shows how filling small holes can be effective. Comparing the distance maps (Fig. 3b,d) calculated from the binary arrays (Fig. 3a,c) reveals the corrupting influence of the small holes. Fig. 3d demonstrates a desired map which is high at the centres of the nuclei and low at the boundaries. Such a desired map will help to split the nuclei at the correct locations.

Each object of *B* which is bigger than *ω*_min_ in size is cropped and processed individually. They are cropped with boxes limited to the objects’ most outer pixels in each direction, called its ‘bounding box’. The original image is also cropped with the same size bounding box as for the object being processed. Then the distance map is calculated for the complement of the binary object inside the bounding box. We incorporate the image intensity into the negative distance map using the same cropped area from the original image. Since the map and intensity do not have the same scale of values, they must be normalized before the summation. The following normalization is defined for an arbitrary array, *A*, to scale its element values to [0, 1].

1 $${\bar{A}}_{n}=\frac{{A}_{n}-\,{\rm{\min }}(A)}{{\rm{\max }}(A)-\,{\rm{\min }}(A)};\,\,{A}_{n} > =0,$$

where *A*_*n*_ and ${\bar{A}}_{n}$ are *n*th element of *A* and its normalized value respectively, *max*(*A*) and *min*(*A*) are the maximum and minimum elements of *A* respectively. When all the elements of *A* are normalized using (1), the output array is denoted by $\bar{A}$.

**Figure 4:**
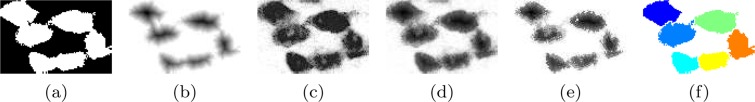
Splitting procedure. (**a**) An object cropped from the binary array *B*. (**b**) $-\bar{D}$, negative normalized distance map of the object. (**c**) $\bar{I}$, normalized intensity inside the object bounding box. (**d**) $S=\bar{I}-\bar{D}$. (**e**) Quantized *cleaned S*. (**f**) Split nuclei.

To explain the splitting process, consider an arbitrary object which is cropped from *B* (Fig. 4a). Its distance map and corresponding cropped intensity are denoted by *D* and *I* respectively (Fig. 4b,c). They are both normalized to [0, 1] range via (1), and denoted by $\bar{D}$ and $\bar{I}$ respectively. Then, we create a new array, $S=\bar{I}-\bar{D}$, by adding the normalized intensity to the negative normalized distance map (Fig. 4d).

The array, *S*, is not yet smooth, i.e. it has many regional minima, therefore applying watershed to it causes oversegmentation. We propose an approach that smooths *S* through a multilevel thresholding and a *cleaning* process. The number of thresholds increases in proportion to the size of the object as described in Algorithm 1. Then *S* is quantized with the specified thresholds. After quantization, there are still unwanted small pieces in each level that must be cleaned. A cleaning loop is started from the first/lowest threshold level to a level immediately before the last/highest level. In each iteration, pieces that are considered as small will be moved to the next higher level. The size of moved pieces in each level increases in proportion to the iteration number as described in Algorithm 1. We apply watershed to quantized *cleaned S* to split the nuclei (Fig. 4e,f). The results are stored in an array, *R* with the same size as the input image. The steps of the splitting procedure is elaborated in Algorithm 1, and illustrated in Fig. 4.

**Figure 5:**
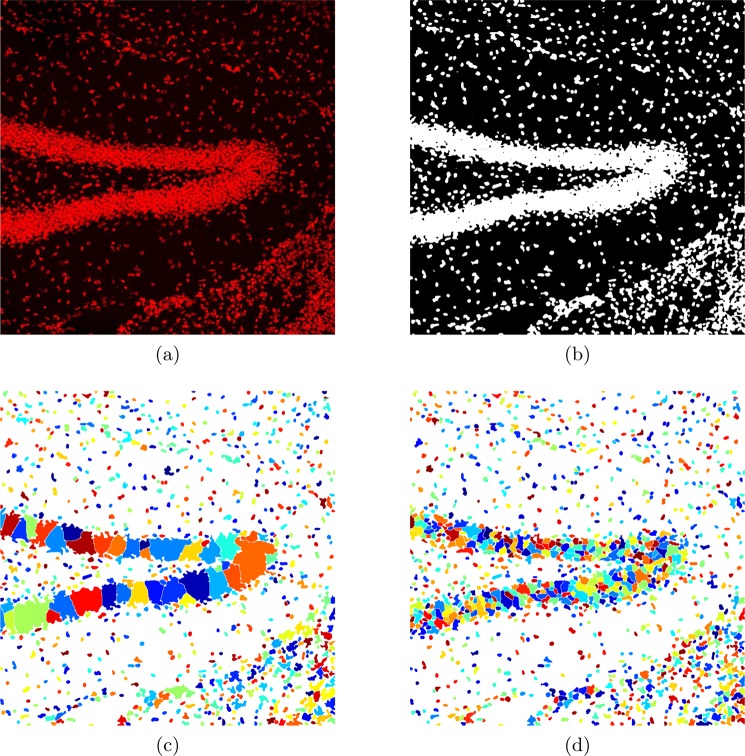
The importance of adding intensity to negative distance map. (**a**) Neuron image (1024 × 1024 pixels showing 1 mm × 1 mm physical size). (**b**) The output of local thresholding step. (**c**) Segmentation result without adding the intensity to the negative of distance map. (**d**) Segmentation result after adding the intensity.

The array, *S*, is formed of two terms: first, the negative distance map in which the lower values the pixels have, the further away they are from the object edges, and second, the distribution of the image intensity. In heavily dense places where many nuclei are clustered, there are many adjacent nuclei connected inside *B* with no gaps (zero pixels/voxels) between them. Therefore the negative distance map is not effective discriminating between adjacent nuclei. However, even a slight change in the intensity (typically darker/lower at the centroid of the nucleus and lighter/higher near the edges) of such nuclei will alter *S* and lead to split them at the correct locations (see Fig. 5).

Fig. 5 demonstrates the importance of adding the intensity to the negative distance map. We have applied our algorithm (with *ω*_min_ = 50 pixels) to an image (1024 × 1024 pixels showing 1 mm × 1 mm physical size) which represents a vast area of heavily clustered neurons (Fig. 5a). The final results of our algorithm, with and without adding the intensity to the negative distance map, are illustrate in Fig. 5c,d respectively. As it is clear from Fig. 5c, the information of the negative distance map alone is not sufficient to separate the nuclei in the heavily clustered places, while adding the intensity has solved this problem (Fig. 5d).

### Model-based merging

The above proposed methods of local thresholding and splitting cluster nuclei precisely segment them and delineate their boundaries. The chance of oversegmentation in *R* is very rare using this splitting approach. Nevertheless, it is useful to include a post-processing step to correct any oversegmentation that may occur^33–36^. Lin *et al*.^34^ have used 8 features (four 3D and four 2D) of the nuclei to define a score based on the nucleus model. The discriminant score was used to recognize oversegmentation and rejoin the qualified nuclei. It has been determined that the most discriminant features are volume and convexity^34^. Convexity is obtained by dividing the area/volume of an object by the area/volume of the smallest convex polygon/polyhedron that contains the object. Therefore convexity is a number less than one, and the closer to one, the more solid the object. We have employed both volume and convexity to build up our merging process.

Every object inside *R* that can potentially attach to others with a common border will be checked. Two merging scenarios might happen in this process: merging between a pair of objects, and a group merging when more than two objects are involved. We first consider the group merging scenario and if it does not happen, then pair merging between objects will be considered. Assume two or more objects can potentially merge together. Two conditions must be satisfied to allow them to merge. First, the convexity of the merged object must be greater than the average convexity of all the individual separate objects. Second, the merged area/volume must remain under a threshold which equals 10 × *ω*_min_. Note that for histopathological cell images, it is reasonable to assume a ratio of minimum to maximum cell size no greater than 10 × *ω*_min_, however, were this method to be applied to other objects, this assumption may need to be re-examined, and a second parameter, maximum object size, introduced.

**Algorithm 1:**
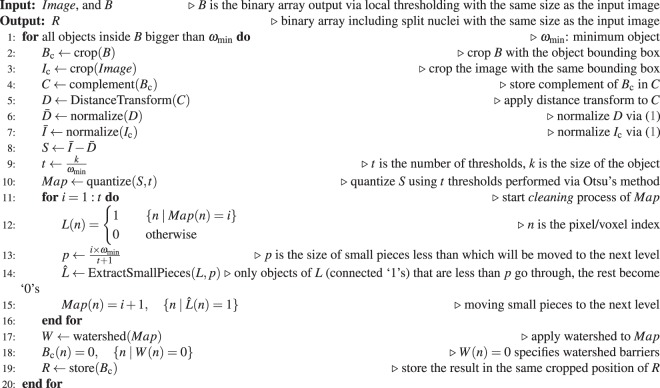
Splitting Clustered Nuclei.

### Model-based correction

The final step in our method is to correct segmented objects based on the nucleus model. We draw boundaries around each object and create local regions using the same approach introduced in Section. Each region includes only one object which will be checked to see whether it qualifies as a nucleus based on its features. Each object would be treated in one of the following ways.

- It will be replaced by a new object, if a new object with larger convexity can be found by thresholding the local region.
- It will be removed if its size is less than *ω*_min_.
- It will be kept as it is, otherwise.

Fig. 5d shows the final output of our method applied to a neuron image. The segmentation result is very satisfying even at heavily clustered area, without any sign of over/undersegmentation in other regions. The whole of the process is automatic with a single parameter, *ω*_min_, set to 50 pixels.

## Results

Four experiments are presented in this section, and the results are compared to those of the state-of-the-art algorithms. First, the ability of our algorithm in recognition of foreground is evaluated through UCSB dataset of breast cancer^38^, stained for hematoxylin and eosin (H&E), and freely available from http://bioimage.ucsb.edu/research/bio-segmentation. The ground truths provided in this dataset are only foreground pixels, without specifying cell boundaries. Therefore, only the ability of foreground recognition is assessed through this dataset. The second and third experiments are performed on BBBC007 dataset of Drosophila Kc167 cells^39^, stained for DNA to label nuclei, and freely available from https://data.broadinstitute.org/bbbc/. In the second experiment, the ability of our method in splitting clustered nuclei is assessed through this dataset, since the outline of each individual cell is provided in the ground truths. The third experiment is an ablation study in which we remove the steps of our algorithm one by one, and then apply it to the whole dataset each time to see how each step affects the overall performance. As the fourth experiment, 3D segmentation ability of our method is evaluated through our own 3D image dataset of mature neurons stained for NeuN.

All the results are evaluated and compared using metrics defined as follows:

2 $$\begin{array}{rcl}p & = & \frac{{t}_{{\rm{p}}}}{{t}_{{\rm{p}}}+{f}_{{\rm{p}}}},\,\,r=\frac{{t}_{{\rm{p}}}}{{t}_{{\rm{p}}}+{f}_{{\rm{n}}}},\\ {f}_{1} & = & \frac{2\times p\times r}{p+r}=\frac{2{t}_{{\rm{p}}}}{2{t}_{{\rm{p}}}+{f}_{{\rm{p}}}+{f}_{{\rm{n}}}},\end{array}$$

in which *p*, *r*, and *f*_1_ are precision, recall (sensitivity), and F1-measure respectively. True positive, *t*_p_, indicates cells/pixels (in pixel-wise assessment) which exist in the ground truth and are correctly found by an automated method. False negative, *f*_n_, indicates cells/pixels exist in the ground truth which are not found by the automated method. False positive, *f*_p_, indicates objects/pixels that are falsely segmented and do not exit in the ground truth.

When the outlines of the cells are provided in the ground truth, the centroids of the segmented nuclei are compared to the centroids extracted from the ground truth outlines. If the Euclidean distance between two centroids (achieved via automated method and extracted from the ground truth) is less than 10 pixels, it will be counted as a true positive, and both centroids will be excluded from further comparison with other remaining centroids. Those centroids that are not matched to any ground truth centroids, are counted as false positives. The remaining unassigned centroids in the ground truth are false negatives.

When the assessment is pixel-wise, two other metrics, ‘accuracy’ and Jaccard index denoted by *a* and *j* respectively, become relevant. These metrics are defined as follows:

3 $$\begin{array}{rcl}a & = & \frac{{t}_{{\rm{p}}}+{t}_{{\rm{n}}}}{{t}_{{\rm{p}}}+{t}_{{\rm{n}}}+{f}_{{\rm{p}}}+{f}_{{\rm{n}}}},\\ j & = & \frac{|A\cap G|}{|A\cup G|}=\frac{{t}_{{\rm{p}}}}{{t}_{{\rm{p}}}+{f}_{{\rm{p}}}+{f}_{{\rm{n}}}},\end{array}$$

in which *t*_p_, *f*_p_, *f*_n_ are as defined previously, and *t*_n_ is true negative and indicates pixels that are background in both ground truth and automated segmentation. *A* and *G* denote automated segmentation and ground truth pixels respectively.

### Segmentation of breast cancer images

58 histopathological images of breast cancer stained for hematoxylin and eosin (H&E), with their ground truths (image patches are roughly 200 × 200) are provided in the UCSB dataset^38^ (image acquisition setting and information are also provided). As the ground truths of this dataset only includes the foreground pixels, and does not specify cell boundaries (i.e. cells are not separated), the only way to evaluate the quality of segmentation is via pixel-wise assessment. There are plenty of studies using this dataset to develop algorithms for segmentation, classification, and etc.^4,18,20,26,40,41^. The evaluation results of the most competitive and recent segmentation techniques performed on this dataset are reported in this section for comparison.

We have applied our method to this dataset, with only one parameter, minimum object, set to *ω*_min_ = 75 pixels. We have also run Segment-nuclei application (Farsight toolkit, release 0.4.5) developed by Al-Kofahi *et al*.^24^ (freely available from http://www.farsight-toolkit.org) on the same dataset, and tuned its parameters via exhaustive search to get the best results (parameters are set to: min-object-size = 75, min-scale = 5, max-scale = 8, xy-clustering-res = 5). A segmentation method based on a deep convolutional network have been recently proposed by Pan *et al*.^9^. They have also used the same dataset to evaluate their method and compare it with others. They have trained their network using 30 images of the dataset. The trained network is then tested by applying it to 28 remaining images. We have also reported their results here for comparison. All methods are applied to the red channel of the colour images, since this channel displays the cells with better quality than grey scale, or other channels.

The results are evaluated via (2) and (3), then the means and the standard errors are presented in Table 1. Our results and Farsight’s^24^ are presented in the first and second row of the table respectively. MI is the multiple image model proposed by Law *et al*.^20^. The last three rows of the table are the results reported by Pan *et al*.^9^. DCN is a deep convolutional network proposed by Pan *et al*.^9^. MSER-based is an approach based on Maximally Stable Extremal Regions proposed by Buggenthin *et al*.^36^. FCM-based approach is based on Fuzzy-C-Means-Clustering proposed by Tang *et al*.^42^. Comparison shows that our method significantly outperforms others in terms of accuracy, precision, and F1-measure. The high recall, but low precision of the Farsight algorithm indicate an overestimation of foreground pixels.

**Table 1:** Evaluation of automated segmentation methods applied to the UCSB dataset of breast cancer.

Methods	Accuracy (%)	Precision (%)	Recall (%)	F1-measure (%)	Jaccard (%)
Ours	**93.93** ± **0.36**	**87.14** ± **0.92**	89.54 ± 0.60	**88.18** ± **0.41**	**78.92** ± **0.65**
Farsight^24^	82.27 ± 1.89	61.03 ± 2.78	**98.23** ± **0.29**	74.15 ± 2.29	60.16 ± 2.66
MI^20^	89.55	—	—	77.33	—
DCN^9^	91.65	84.89	80.84	82.34	—
MSER-based^36^	84.63	61.37	88.57	72.50	—
FCM-based^42^	86.60	64.04	94.49	76.34	—

Metric columns show means (±standard errors).

**Figure 6:**
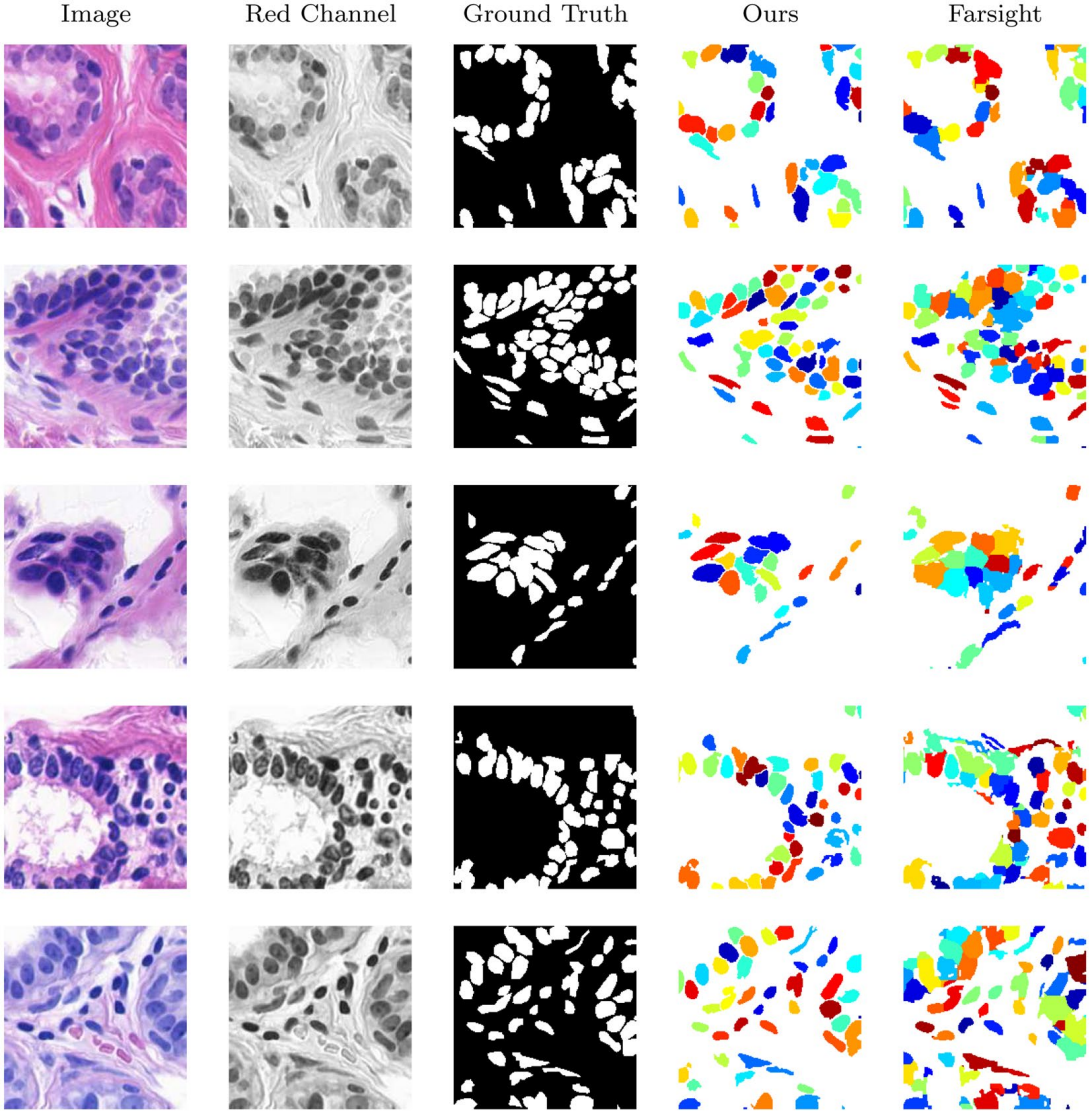
Segmentation of breast cancer images.

Five original images of this dataset, their red channels and ground truths, our segmentation results, and Farsight’s are illustrate in Fig. 6. Colourful representation of the cells in which each cell has its own colour, demonstrates the high strength of our method in splitting clustered cells. Comparison shows our results are very close to the ground truths and outperform Farsight.

### Experiments on Drosophila Kc167 cell dataset

In this section, first we evaluate the strength of our method in splitting clustered nuclei, and then perform an ablation study of our algorithm.

#### Segmentation of Drosophila Kc167 cells

The BBBC007 dataset of Drosophila Kc167 cells stained for DNA to label nuclei, includes 16 image patches (their sizes are roughly 400 × 400) of the cell nuclei and the ground truths provide outlines of the nuclei^39^.

The algorithms are applied to this dataset with the best tuned parameters (obtained via exhaustive search to get the best results) as follows: 1- ours with the minimum object set to *ω*_min_ = 30 voxels; 2- Segment-nuclei^24^ (Farsight toolkit, release 0.4.5), available from http://www.farsight-toolkit.org, its parameters were set to: min-object-size = 30, min-scale = 5, max-scale = 8, xy-clustering-res = 5; 3- Objects-counter^43–45^ (release 2.0.1, Fiji distribution plug-in), available from https://imagej.net/Welcome, its parameter was set to: size-filter-min = 30; 4- Label-objects^46^ (release 1.3, Vaa3D plug-in) available from http://home.penglab.com/proj/vaa3d/Vaa3D/, its parameter was set to: small-components = 30.

The results are assessed and compared via the metric defined in (2). The means and standard errors of the evaluation results, and the average running times are presented in Table 2. The comparison shows our method outperforms others in terms of precision and F1-measure. The average running times are close to 1 second and there is not much difference between methods for this dataset, since the patch sizes are small (roughly 400 × 400 pixels). To compare the running times of different algorithms, they will be applied to a dataset which includes large 3D images in the next experiment.

**Table 2:** Evaluation of automated segmentation methods applied to the BBBC007 dataset of Drosophila Kc167 cells.

Methods	Precision (%)	Recall (%)	F1-measure (%)	$\bar{t}$
Ours	**95.26** ± **0.82**	97.11 ± 1.07	**96.11** ± **0.71**	1.0
Farsight^24^	79.10 ± 1.67	**97.56** ± **0.55**	87.20 ± 1.06	0.7
Fiji^43–45^	94.92 ± 1.38	87.53 ± 3.83	90.59 ± 3.00	1.0
Vaa3D^46^	85.57 ± 3.48	68.35 ± 4.63	75.51 ± 4.41	1.1

Metric columns show means ± standard errors. Average running time, $\bar{t}$, is in seconds for the image patch sizes roughly 400 × 400. System specifications: Windows 10 running on Dell, Intel core i7 with 16 GB memory.

**Figure 7:**
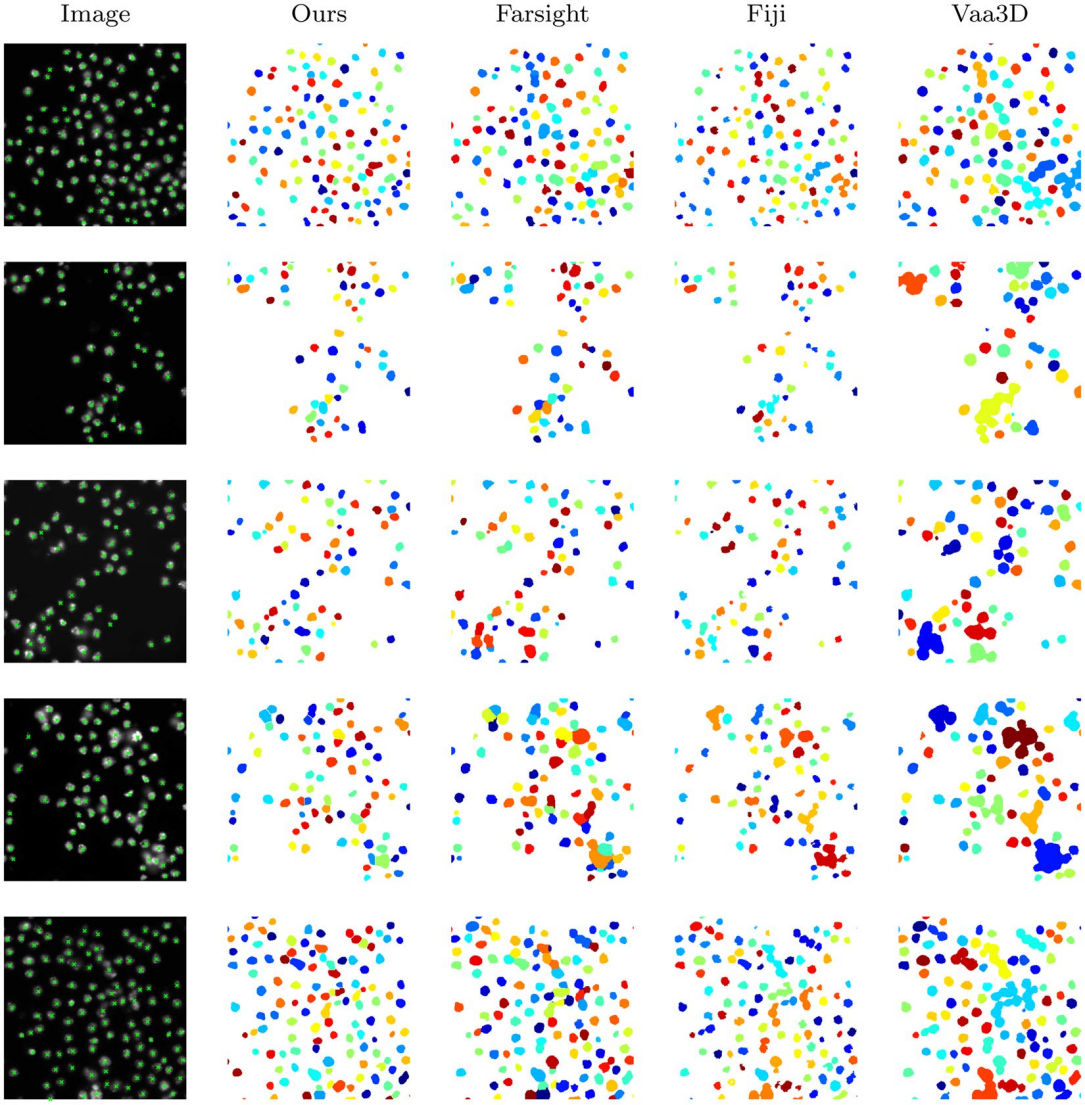
Segmentation of Drosophila Kc167 cells.

Five images of this dataset and their segmentation results via the aforementioned algorithms are illustrated in Fig. 7. The centroids of the nuclei have been overlain on the original images and depicted as green crosses. Segmented nuclei are assigned random colours to demonstrate the ability of algorithms in splitting the clustered nuclei. As it can be seen our method has performed very successfully in separating touching nuclei and delineating their boundaries, and outperforms the others.

#### Ablation study of our algorithm

**Figure 8:**
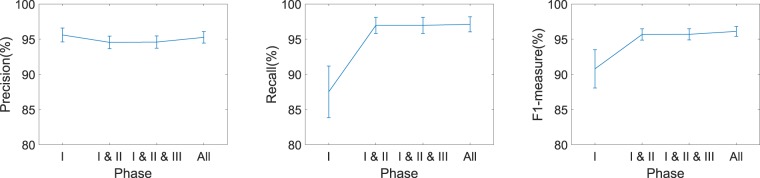
Ablation study of the proposed method. Graphs show mean values of the metrics including standard error bars.

An ablation study is performed on our algorithm using the same dataset to see how each phase of our method contributes to its overall performance. As it is elaborated in the Method section, and illustrated in Fig. 1, our proposed algorithm comprises four main consecutive phases, I: adaptive thresholding, II: splitting clustered nuclei, III: model-based merging, and IV: model-based correction. The results of our whole algorithm running through the dataset are presented in previous section. We have performed the same task three more times after removing phase IV; phase IV and III; and phase IV, III and II. The results are presented in Table 3 and illustrated in Fig. 8. As it is clear from both table and figure, phase II significantly improves the output of phase I in terms of the recall and thus F1-measure by splitting clustered nuclei. For this dataset, the least effective is phase III which shows merging process rarely happened. In general phase III has the least impact on performance of all the phases, however since the impact is always positive, this phase is worth retaining in our algorithm. Overall, the significant improvement in recall with the precision almost steady leads to an improvement of F1-measure with the addition of each phase.

**Table 3:** Ablation study of our proposed method. Metric columns show means ± standard errors.

Phase	Precision (%)	Recall (%)	F1-measure (%)
I	**95.60** ± **0.99**	87.51 ± 3.67	90.79 ± 2.72
I & II	94.55 ± 0.89	96.96 ± 1.44	95.67 ± 0.80
I & II & III	94.59 ± 0.88	96.96 ± 1.14	95.69 ± 0.80
All	95.26 ± 0.82	**97.11** ± **1.07**	**96.11** ± **0.71**

### Segmentation of 3D neuron dataset

As the fourth experiment, 3D segmentation ability of our method is evaluated using a 3D image dataset of mature neurons stained for NeuN to label nuclei, imaged in our lab. All experiments were approved by the University of Newcastle Animal Care and Ethics Committee, and conducted in accordance with the New South Wales Animals Research Act and the Australian Code of Practice for the use of animals for scientific purposes.

Immunohistochemistry is as follows: free-floating, 30 μm PFA fixed brain sections were immuno-stained using standard protocols, a primary antibody mouse anti-NeuN (#MAB377, Millipore, 1:500) followed by secondary antibody (Alexa-Fluor 594, goat anti-rabbit # R37117). Confocal images have been taken on a Leica TCS SP8 confocal microscope with a Leica HC 25x/0.95 water, Leica HC PLC APO 40x/1.30 or 63x/1.40 OIL objective.

The prepared dataset for analysis includes 8 image patches, each with the dimensions of 352 × 352 × 12 voxels spanning 30.75 μm × 30.75 μm × 16.51 μm physical size of the section. The centroids of the nuclei have been manually annotated to provide ground truth data needed to evaluate the performance of several algorithms. The quality of annotations have been examined by an expert in neuroscience.

Our method in addition to two other state-of-the-art methods have been applied to this dataset. The algorithms and the best tuned parameters found via exhaustive search to achieve the best results, are as follows: 1- ours with the minimum object set to *ω*_min_ = 4000 voxels; 2- Segment-nuclei^24^ (Farsight toolkit, release 0.4.5), its parameters were set to: min-object-size = 4000, min-scale = 25, max-scale = 30, xy-clustering-res = 5; 3- Objects-counter^43–45^ (release 2.0.1, Fiji distribution plug-in), its parameter was set to: size-filter-min = 4000. We have also applied Label-objects^46^ (release 1.3, Vaa3D plug-in), but were unable to segment the neurons.

Table 4 presents the results of the aforementioned methods assessed via three metrics defined in (2). As it is clear from the table, our proposed method has performed well. Also, the average computational times provided in the table clarify that this performance does not come of the cost of additional complexity.

**Table 4:** Evaluation of automated segmentation methods applied to our 3D neuron dataset.

Methods	Precision (%)	Recall (%)	F1-measure (%)	$\bar{t}$
Ours	**92.71** ± **4.84**	**94.15** ± **2.95**	**92.53** ± **2.62**	1.29
Farsight^24^	89.58 ± 5.16	91.03 ± 3.64	89.21 ± 2.82	2.85
Fiji^43–45^	51.39 ± 6.94	37.40 ± 9.02	41.15 ± 7.94	79.55

Metric columns show means ± standard errors. Average running time, $\bar{t}$, is in seconds for the image patch sizes 352 × 352 × 12. System specifications: Windows 10 running on Dell, Intel core i7 with 16 GB memory.

**Figure 9:**
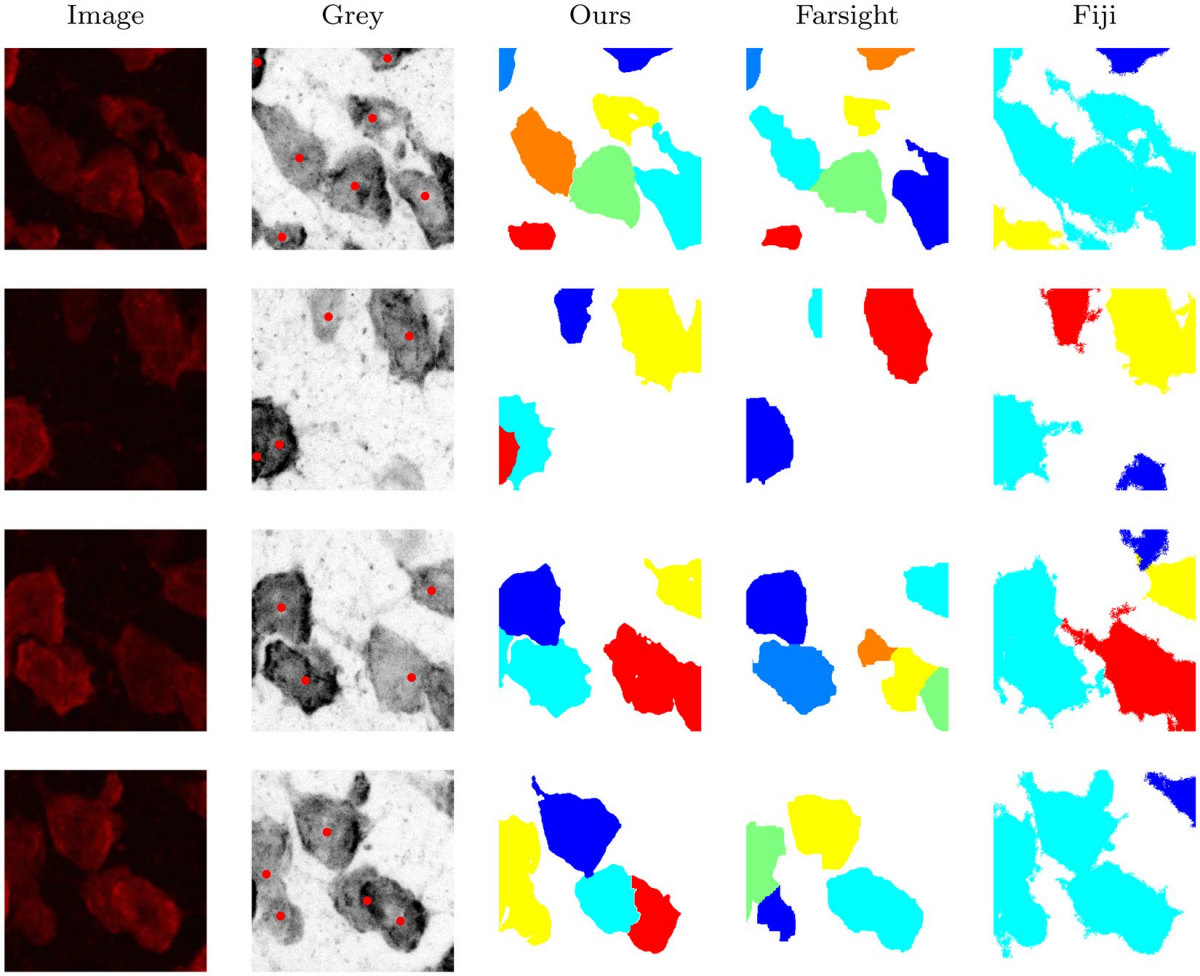
Segmentation of our 3D neuron dataset.

To visualize this 3D dataset and some of the segmentation results, x-y views of maximum intensity projection of the images are employed in Fig. 9. This figure includes five original images from the dataset, the grey scale versions of them including manually annotated centroids (red dots). Note, cells underneath may not be properly seen in the x-y view since they might be covered by others on top slices. However, they have been considered in the manual annotation, and their presence are clarified here using the red dots. The respective segmentation results achieved via our method, Farsight, and Fiji are also illustrated in Fig. 9. It can be seen that our method is very successful in separating touching nuclei and delineating their boundaries. Farsight suffers from some undersegmentation when the nucleus clump is dense, and oversegmentation when the stain is weak and uneven. Also, Fiji’s results are very poor, and it has obviously failed to separate the nuclei.

## Discussion

In this paper, a novel method of cell nucleus segmentation has been proposed, which is applicable to variety of histopathological images obtained via different staining methods. The initial classification of the image foreground was performed by locally adaptive thresholding on watershed regions. To split clustered nuclei, a combination of their intensity and distance map were employed, and then smoothed through a multilevel thresholding and a cleaning process. The application of watershed to the smoothed arrays split the clustered nuclei at correct locations and produced satisfying results. A model-based merging was employed to eliminate oversegmentation. We finalized our algorithm with a model-based correction step to improve the result and eliminate small objects.

One of the advantage of our method is that the image intensity is considered during the splitting process. This idea helps to separate heavily clustered nuclei at correct locations. The simplicity of the application with no training required, high speed, accuracy and level of automation with only a single parameter required, are the other benefits of our method.

As the experimental results, we applied our method to three histopathological image datasets of breast cancer, Drosophila Kc167 cells, and neuron nuclei. The evaluation of the results shows the strength and high efficiency of our method which outperform that of the state-of-the-art methods in terms of accuracy, precision, F1-measure, and computational time.

Morphological study of the cells, e.g. feature extraction and classification of cell population, and cell movement tracking in time lapse image sequences, need cell nucleus segmentation as a fundamental step. Therefore, this study builds a perfect basis for a comprehensive study of cell population as a future work.
